# Flying bats use serial sampling to locate odour sources

**DOI:** 10.1098/rsbl.2021.0430

**Published:** 2021-10-20

**Authors:** Alyson F. Brokaw, Evynn Davis, Rachel A. Page, Michael Smotherman

**Affiliations:** ^1^ Interdisciplinary Program in Ecology and Evolutionary Biology, Texas A&M University, College Station, TX, USA; ^2^ Department of Biology, Texas A&M University, College Station, TX, USA; ^3^ Kreiger School of Arts and Sciences, Johns Hopkins University, Baltimore, MD, USA; ^4^ Smithsonian Tropical Research Institute, Balboa, Panama

**Keywords:** bats, search strategies, foraging, olfaction, olfactory tracking

## Abstract

Olfactory tracking generally sacrifices speed for sensitivity, but some fast-moving animals appear surprisingly efficient at foraging by smell. Here, we analysed the olfactory tracking strategies of flying bats foraging for fruit. Fruit- and nectar-feeding bats use odour cues to find food despite the sensory challenges derived from fast flight speeds and echolocation. We trained Jamaican fruit-eating bats (*Artibeus jamaicensis*) to locate an odour reward and reconstructed their flight paths in three-dimensional space. Results confirmed that bats relied upon olfactory cues to locate a reward. Flight paths revealed a combination of odour- and memory-guided search strategies. During ‘inspection flights’, bats significantly reduced flight speeds and flew within approximately 6 cm of possible targets to evaluate the presence or absence of the odour cue. This behaviour combined with echolocation explains how bats maximize foraging efficiency while compensating for trade-offs associated with olfactory detection and locomotion.

## 1. Introduction

Tracking odours to their source is a complex task that depends upon the cognitive reconstruction of spatio-temporal odour gradients in the environment and is sensitive to locomotor speed [1–4]. Terrestrial animals display a suite of strategies that increase their chance of detecting an odour plume, including reducing travel speed, increasing sampling rate and moving in an undulating pattern [5–8]. Flying animals move quickly through complex, turbulent environments, which imposes serious constraints on their ability to detect odour gradients and resolve fluid movement direction [9]. The mechanisms flying vertebrates use to compensate for these challenges are still unknown. Here, we provide the first description to our knowledge of how flying bats search for an attractive odour source.

Most work on olfactory tracking strategies in flight has focused on invertebrates, which both follow odour plume gradients in flight (i.e. moths, [10]) and rely on directional and spatial memory (i.e. tsetse flies, [10,11]). While some seabirds and vultures may use long-distance olfactory cues to locate odour sources [12–14], the behaviour of flying vertebrates has not been tested empirically. Neotropical fruit- and nectar-feeding bats rely upon echolocation for navigation but are also known to use olfactory cues while foraging [15,16] and are highly sensitive to some fruit-typical odours [17]. Previous experimental research demonstrated that fruit bats can detect and follow odour concentration gradients [18], particularly when crawling [19], but not how their olfactory search strategies might differ from or complement echolocation-based searches.

Here, we describe the olfactory foraging strategies used by the Jamaican fruit-eating bat (*Artibeus jamaicensis*), which feeds on a variety of fruits, including banana [20,21], and demonstrate preferences for fruit odours when foraging [22–24]. We confirmed that bats could successfully locate a food reward by odour cues, then used three-dimensional path reconstruction to characterize their tracking strategy in flight. If bats used odour gradients in flight to solve an olfactory localization task, we expected that individuals would correctly investigate and choose the odour more often than predicted by chance [1,14]. Alternatively, the constraints of flight may force bats to rely on other strategies, such as serial sampling or route-following [25,26], wherein bats are motivated by the presence of an attractive odour but need to sample each site until the odour source is located.

## 2. Material and methods

### (a) Animal capture and care

Behavioural experiments were conducted at the Smithsonian Tropical Research Institute in Gamboa, Panama (9°07'14.5″ N, 79°42'08.2″ W). Bats were captured using mist-nets in Soberanía National Park, Panama. Only adult, non-reproductive individuals were kept for experiments, housed together in holding cages (1 × 1 × 2 m) between nights. During the first 24 h, bats were provided water and banana *ad libitum*. Olfactory stimuli were always presented to bats on wooden platforms (1.2 m high).

### (b) Experimental set-up and procedure

We measured olfactory localization behaviour in flying bats using a multiple-choice assay with standard operant procedures (electronic supplementary material, movie S1). Prior to experimental trials, bats were released individually into the experimental chamber (5 × 5 × 2.5 m flight cage) and offered banana pieces placed on five platforms across the back of the room. Individuals that spontaneously consumed banana from the platforms during this 30 min period were retained for subsequent experiments (male = 20, female = 16). All trials were carried out in near-darkness, illuminated using infrared LED lights and synchronously recorded using digital video cameras.

**Figure 1.**
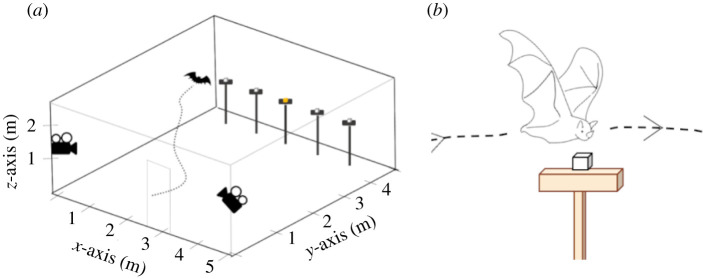
(*a*) Diagram of the experimental area, showing the position of the cameras and the stimulus platforms. Cubes on the platforms represent S+ (yellow) and S− (white). (*b*) Illustration of an investigation event.

The order of individuals and of experimental treatment was randomized within and across nights to reduce potential confounding effects of learning and to sustain motivation by providing real banana every third or fourth trial. The location of the reward (S+) was pseudo-randomized to ensure that it was placed on each platform at least once, and its position was not repeated consecutively between trials. For additional details, see figure 1*a* and the electronic supplementary material.

#### (i) Experiment 1: localization of food reward using odour

We first confirmed if bats could successfully localize an odour reward (banana, S+) in the experimental arena. One banana piece (approx. 2.5 × 2.5 × 1.5 cm) supplemented with 0.1 ml of 100% food-grade banana baking emulsion was placed on a random platform in the flight cage. The other four platforms held a wet cosmetic sponge (S−) cut into the same shape as the banana. Trials started when a bat was released into the chamber and continued until the bat landed on the correct platform. If bats failed to locate the banana within 20 min, the trial was ended and a ‘no-choice’ result was logged.

#### (ii) Experiment 2: role of echoacoustic cues in reward localization

Bats may be able to distinguish banana from banana-shaped sponges based on echolocation, since reflected echoes might contain discriminable acoustic features. In Experiment 2, we tested if bats could successfully locate the odour (S+) when all five stimuli were the same material. For S+, an odour cue was prepared by soaking the sponge in a banana–sugar mixture (see electronic supplementary material). Controls and experimental procedure were the same as described above.

### (c) Behaviour analysis

We determined that a bat had made a choice when it landed on a platform. Trials were scored a ‘success' when bats correctly chose the platform containing S+. For each trial, we documented the total number of investigation events, defined as a flight near and across a platform, accompanied by the bat lowering its head or directing its nose towards the platform (figure 1*b*). We also counted the total number of platforms investigated, excluding repeated visits (unique platform investigations). Landing behaviours were not considered investigation events. Behaviours were manually scored by the same individual (A.F.B.), using EthoVision XT 13 and BORIS v. 7.9.16 [27].

Preliminary observations indicated that investigation events played a central role in the bat olfactory search strategy. To characterize them further, we reconstructed the three-dimensional flight paths for a subset of trials from nine bats using EthoVision XT 13 Track3D software (figure 1*b*; see electronic supplementary material). We compared instantaneous flight speed during an inspection event with the flight speed of a pseudo-random control when the bat was flying straight across the arena. Since bats typically flew directly over platforms, we used vertical distance during inspections to calculate a minimum distance from the stimulus.

### (d) Statistical analysis

Performance for each experiment was summarized as the percentage of trials in which the bat correctly chose the platform containing the odour stimulus (S+). We used one-tailed binomial tests to assess if individual bats performed better than chance (20%) during the trials. If bats are following an odour plume to select the correct platform, we expected that bats would first investigate the S+ platform more often than expected by chance. If bats used odour to make their final choice, we predicted that the last platform investigated would be the same as their final choice. Therefore, we used one-tailed binomial tests to assess if individuals performed better than chance (20%) in their first inspection and last inspections of a platform.

Next, we examined the distribution of total number of investigation events and unique platform investigations in successful trials. To test if there was a common number of unique platform investigations, we used a repeated-measures ANOVA (‘lme’ in package ‘nlme’, Pinheiro *et al*. [28]), setting number of investigated platforms as a categorical variable (0–5). We then applied Tukey's *post hoc* comparisons, adjusted for multiple comparisons (‘glht’ in package ‘multcomp’ [29]). Finally, we used paired *t*-tests to compare the average speed of bats investigating the platforms with the average speed of control events in the same flight path. All analyses were carried out using R [30] and RStudio [31].

## 3. Results and discussion

### (a) Use of olfactory cues in foraging

Experiments 1 and 2 confirmed that flying bats successfully located S+ by olfactory cues. All bats demonstrated a success rate for locating the banana reward significantly higher than expected by chance (one-tailed binomial test, *p* < 0.05; electronic supplementary material, table S1). When we controlled for echolocation cues (Experiment 2), bats located the odour reward in 87.3% of the trials (table 1) and all but two individuals demonstrated a success rate significantly higher than expected by chance (one-tailed binomial test, *p* < 0.05; electronic supplementary material, table S2). These results are consistent with previous studies demonstrating the importance of olfactory cues in food evaluation and selection of fruit- and nectar-feeding bats [15,16,32–34].

**Table 1.** Summary of bat success in Experiments 1 and 2. Success is defined as bat landing on S+ platform. ‘First adjacent’ includes investigations of platforms adjacent to S+, while ‘last investigated’ indicates if the bat investigated the S+ platform before making the correct choice (successful trials only).

behaviour	banana ± s.e.m. (242 trials, *n* = 33 bats)	odour ± s.e.m (142 trials, *n* = 21 bats)
choice	87.8 ± 1.7%	87.3 ± 3.2%
first investigated	23.4 ± 3.4%	24.7 ± 4.3%
first adjacent	59.2 ± 2.8%	50.4 ± 3.7%
last investigated	67.8 ± 3.9%	61.3 ± 5.3%

### (b) Olfactory search strategies in flight

If bats used odour plumes to locate attractive odour sources, we predicted they would approach those platforms early in the search. Instead, we found that bats did not perform better than random chance at finding the S+ platform on their first inspection in either experiment (one-tailed binomial tests, *p >* 0.05). We then considered whether bats might use odour plumes to narrow down the general location of S+ to correct or adjacent platforms. When investigations of platforms adjacent to the platform holding S+ were included, bats were only slightly more successful. The location of last inspections and final choices matched in over 60% of trials (table 1), confirming that close inspections are important in finding S+.

**Figure 2.**
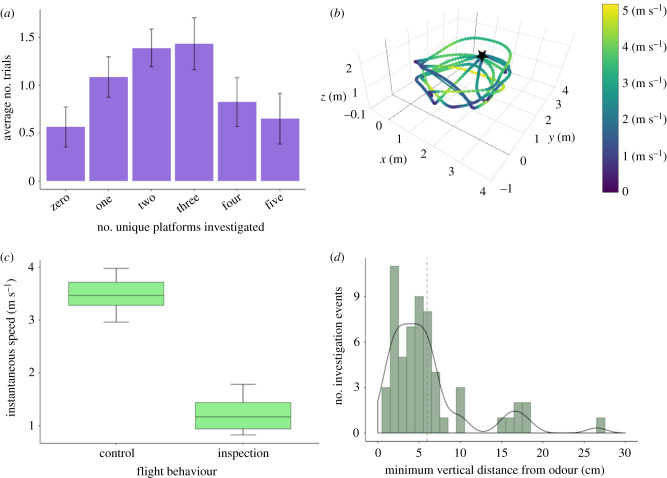
(*a*) Comparison of the number of unique platforms investigated by bats before landing (successful trials, Experiment 2). Error bars indicate within-subject standard error. (*b*) Example three-dimensional flight path reconstructions of a bat navigating to the odour cue. Star indicates the location of odour. (*c*) Boxplots showing average flight speed for control and inspection timepoints. (*d*) Histogram of minimum vertical distance from top of the platform for inspection events (40 trials, *n* = 9 bats). Dashed line represents the mean.

These results suggest that bats were unlikely to be mapping odour plumes to locate the odour source. Instead, bats appeared to use a serial sampling strategy, investigating several potential locations up close before making their final decision. On average, bats performed three to four investigations per trial (weighted average = 3.71), including repeat visits. Focusing on unique investigations, bats typically inspected at least two different platforms before choosing (weighted average = 2.32). Bats investigated two or three different platforms significantly more often than investigating zero, one, four or five platforms (figure 2*a*; repeated-measures ANOVA, *F* = 2.47, *p* = 0.03).

This multi-sampling approach is consistent with observations of foraging bats in the wild. Field studies in Central America suggest that *A. jamaicensis* use flyways during foraging [35,36], perform ‘scouting’ behaviours of fruit trees in their home range [36], and frequently return to the same tree multiple nights in a row [36,37]. Spatial memory contributes to fruit bat foraging behaviour and may even overshadow sensory cues such as odours or acoustics [38]. Tracking studies in Egyptian fruit bats (*Rousettus aegyptiacus*) support the use of spatial memory foraging, finding no correlation between wind direction and bat movements that would be indicative of odour plume following at large scales [39,40].

### (c) Characterization of investigation manoeuvres

We reconstructed the flight paths of successful trials from nine individuals in Experiment 2 (figure 2*b*). Bats moved significantly slower when inspecting the platforms compared with controls across trials (paired *t*-test, *t* = 24.85, *p* < 0.001; figure 2*c*). During inspections, bats closely approached the platforms, averaging an estimated minimum vertical distance of 5.8 cm (±0.7 cm), with most inspections at or below 10 cm (figure 2*d*). This measurement may slightly underestimate total distance since it does not account for bat distance from the platform in the *x* or *y* direction, but it is well within the approximately 30 cm detection threshold found for crawling fruit bats [19]. These behaviours are similar to those of dogs, which adjust speed and posture during odour trail tracking [6,41].

*Artibeus jamaicensis* produce broadband, frequency-modulated calls of low amplitude from their noses [15,42,43]. Acoustic recordings confirmed that the bats continuously echolocated throughout the olfactory task, but the stimuli in Experiment 2 differed only in the presence of an odour cue. By slowing down and closely approaching the platforms, bats may be better able to discern olfactory cues. It does not appear that nasal pulse emission interfered with olfactory sampling, but our experiments could not address how echolocation and sniffing are coordinated during flight.

We propose that bats rely on spatial memory and echolocation to orient within the experimental area, then use a combination of echolocation and olfaction to make a decision about the position and composition of a target. This strategy extends previous observations that bats use odours to detect the presence of potential food and use echolocation to precisely localize fruit at close range [15,16]. Since flying bats move faster than mice or other terrestrial animals and the location of individual trees does not change, quickly sampling a few potential locations and using odour to make a final selection may be more efficient than trying to follow unpredictable odour plumes in a cluttered environment.

## 4. Conclusion

Overall, our results provide an extended hypothesis of the strategies fruit bats use to locate fruit on a tree. Though we cannot entirely rule out plume tracking, it appears that the Jamaican fruit-eating bat, *A. jamaicensis*, combines echolocation, serial sampling and olfactory cues for the localization of a reward. Although these results may be biased by the small-scale nature of our experiments, they are consistent with field observations in this and other fruit-eating bats [44–46]. Advances in tracking technology will allow further examination of how bats integrate cognitive and sensory behaviours at landscape scales. The use of olfactory cues for foraging in non-frugivorous bats is not well understood, with limited experimental evidence for olfactory foraging in carnivorous and sanguivorous bats [47,48]. Future work could investigate if these bats use similar olfactory search behaviours. Understanding how bats locate resources is important for predicting how landscape changes (such as habitat fragmentation) may affect populations, particularly in tropical forest ecosystems [49,50].

## Data accessibility

Data and R code used in this manuscript are publicly available on figshare [51]. The data are provided in the electronic supplementary material.
